# The potential role and value of vitamin D in the treatment of tuberculosis

**DOI:** 10.3389/fcimb.2025.1654860

**Published:** 2026-01-14

**Authors:** Meng Zeng, Jiyu Ran, Yun Luo, Xue Zhou, Yan Hu, Xiangyu Tian

**Affiliations:** 1 Department of Clinical Laboratory, The Affiliated Yongchuan Hospital of Chongqing Medical University, Chongqing, China; 2 Tuberculosis Reference Laboratory, Chongqing Tuberculosis Control Institute, Chongqing, China; 3 Department of Clinical Laboratory, Chongqing Yongchuan District Ji′ai Hospital, Chongqing, China

**Keywords:** infection, *Mycobacterium tuberculosis*, treatment, tuberculosis, vitamin D

## Abstract

Tuberculosis remains a prevalent and serious chronic bacterial infection worldwide. Despite significant advancements in TB treatment in recent years, it continues to pose a major public health challenge. The onset and progression of TB are closely associated with individuals who are immunocompromised, as most patients also present comorbidities such as HIV, diabetes mellitus, and nutritional deficiencies. Consequently, the development of new, non-toxic immunomodulatory drugs or treatment strategies may offer viable solutions to these issues. Vitamin D not only plays a crucial role in regulating calcium and phosphate metabolism while maintaining bone health but is also a key regulator of the innate immune response against microbial infections. Furthermore, many tuberculosis patients exhibit low levels of vitamin D; thus, vitamin D may represent an important resource for enhancing immune responses against *Mycobacterium tuberculosis* infections. This review discusses the immune response mechanisms, vitamin D synthesis processes, and metabolic pathways activated in hosts following infection with *M. tuberculosis*. It emphasizes how vitamin D contributes to immune regulation and its potential role in combating *M. tuberculosis* infections within the human body. This literature review aims to provide theoretical support for developing new drugs and treatment strategies for clinical management of anti-*M. tuberculosis* infections.

## 1 Introduction

Tuberculosis is a chronic infectious disease caused by the infection of *Mycobacterium tuberculosis*. The lungs are the most common site of infection; however, the bacteria can also invade other organs such as the kidneys, bones, and brain, leading to extrapulmonary tuberculosis. According to the data from the World Health Organization (WHO), approximately 10 million new cases of tuberculosis are reported globally each year, with an incidence rate of about 130 per 100,000 individuals (Dheda et al., 2022), TB remains one of the top ten causes of death worldwide (Stein, 2023). The incidence rates of TB vary significantly across different countries and regions. This disparity is particularly pronounced in low- and middle-income countries where the burden imposed by TB is substantial. Regions such as Africa, Southeast Asia, and the Western Pacific account for over 80% of global TB cases. Factors such as HIV infection, malnutrition, diabetes mellitus, smoking, and others considerably increase the risk of developing TB (Boadu et al., 2024; Meintjes and Maartens, 2024). Currently available clinical treatments for TB include rifampicin, isoniazid, fluoroquinolone antibiotics, pyrazinamide, and ethambutol (Vanino et al., 2023), despite significant advancements in diagnosis and treatment due to these medications and improvements in healthcare standards over recent years—challenges remain due to several factors: 1) The lengthy treatment duration (6–9 months) of TB; 2) Significant side effects including liver injury, gastrointestinal reactions, neuropsychiatric symptoms, allergies, rashes, slight vision loss, etc.; 3) The limited efficacy of these drugs (Singh, 2024); 4) More and more drug-resistant strains are emerging. These challenges create multiple obstacles for treating patients with tuberculosis which exacerbates the prevention and control efforts against *M. tuberculosis*. Consequently, there has been a growing focus on researching new drugs or treatment strategies that may offer adjunctive benefits in managing this disease effectively.

Vitamin D is a fat-soluble steroid vitamin that is essential not only for maintaining bone health but also for regulating immune function, cell differentiation, and anti-inflammatory responses (Bikle, 2022). An increasing body of research indicates that vitamin D may play a significant auxiliary role in the prevention and treatment of tuberculosis (Facchini et al., 2015; Tamara et al., 2022). This article aims to summarize the potential roles and mechanisms of vitamin D in TB management, with the objective of providing data support for the development of new anti-tuberculosis drugs and innovative treatment strategies.

## 2 The immune response of the body after infection with *M. tuberculosis*

**Figure 1:**
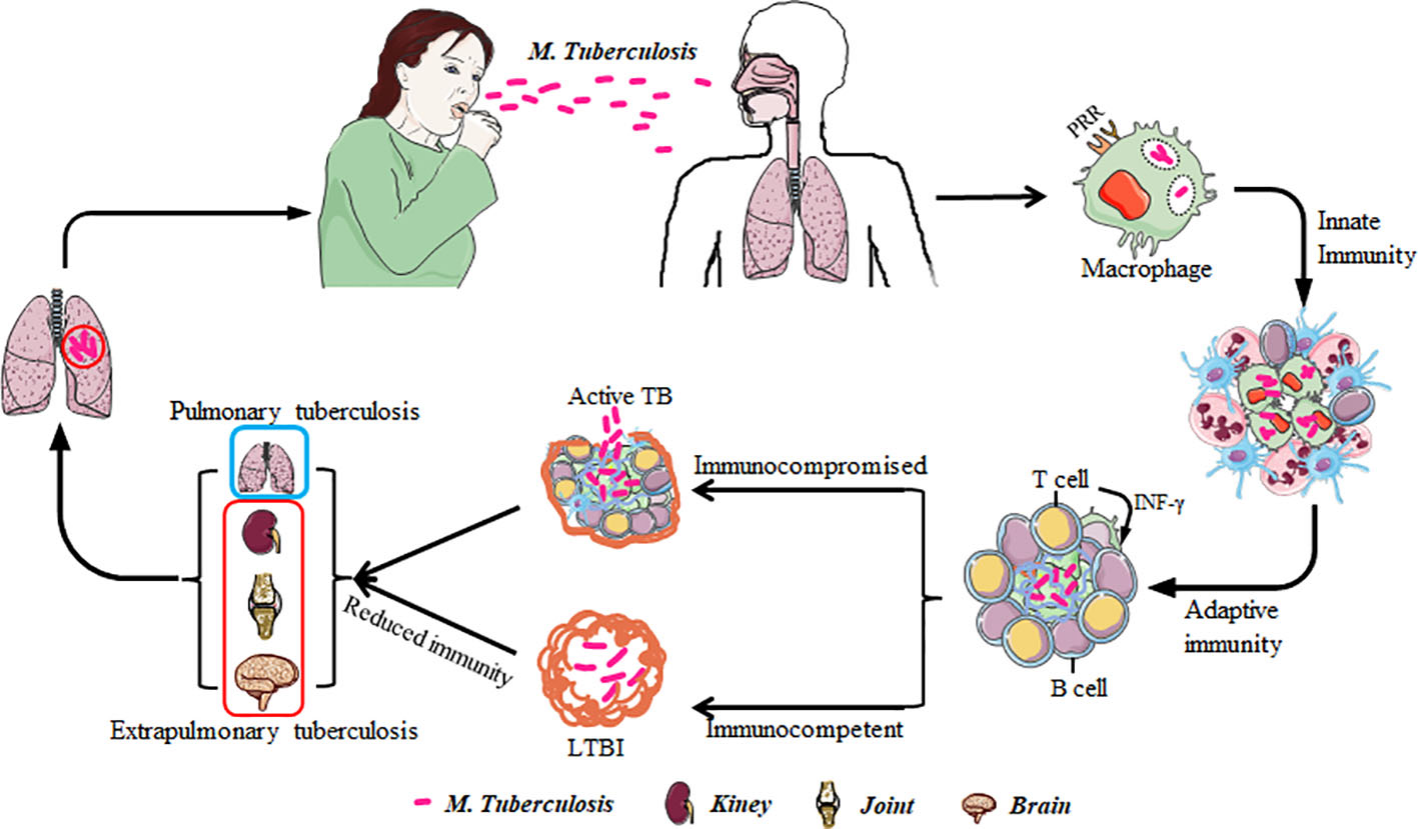
The transmission and infection routes of *M. tuberculosis*. *M. tuberculosis* is transmitted into the human body through the respiratory tract and is recognized and phagocytosed by lung macrophages, activating innate and adaptive immune responses. When the immune function of the person is normal, *M. tuberculosis* lies dormant in the human body and becomes a latent infected person. When the immune function of the infected group is low, *M. tuberculosis* multiplies into TB patients and further spreads through the respiratory tract. Besides the common pulmonary infections, there are also extrapulmonary infections such as renal TB and bone TB.

*M. tuberculosis* is an intracellular pathogen transmitted via aerosolized droplets, initiating infection in the lungs (Eletreby et al., 2024). Upon entry, the innate immune response, involving macrophages, dendritic cells, monocytes, and neutrophils, is activated. These cells phagocytose *M. tuberculosis*, representing the initial defense against the pathogen. After the inhaled *M. tuberculosis* reaches the alveoli and is recognized by immune cells, two primary outcomes will occurred: one is that some pathogens are directly engulfed and eliminated by immune cells, and then another part of pathogens survive and multiply in macrophages under the attack of immune cells (Cohen et al., 2018), and form granulomas with the participation of other immune cells such as neutrophils, natural killer cells, DC cells, T cells, etc., tightly wrapping *M. tuberculosis* and preventing its further spread in the body (Cohen et al., 2022). What’s more, *M. tuberculosis* may disseminate via the lymphatic or circulatory systems, leading to extrapulmonary TB in sites such as the lymph nodes, bones, kidneys, or brain (**Figure 1**).This transmission characteristic of *M. tuberculosis* makes TB not only limited to the lungs, but also may affect multiple organ systems, increasing the complexity of the disease and the difficulty of treatment. In addition, *M. tuberculosis* can also enter the human body through digestive tract or skin wounds, but these modes of transmission are relatively rare (Baykan et al., 2022).

The entry of *M. tuberculosis* into the body can trigger a series of complex immune responses. Macrophages, as the earliest defenders against *M. tuberculosis* infection, not only serve as a bridge between innate and adaptive immune responses, but also act as host cells after the infection of *M. tuberculosis*. Therefore, macrophages play a crucial role in clearing *M. tuberculosis* infection (Cohen et al., 2018; Rienksma et al., 2019). According to literature reports, after macrophages recognize and swallow *M. tuberculosis* through multiple receptors (Toll-like receptors/C-type lectin receptors) receptors, and then present the pathogen antigens to T cells in the form of antigen peptide-Major Histocompatibility Complex (Basu et al., 2007; Yang et al., 2018). At the same time, macrophages regulate the secretion of the inflammatory factor tumor necrosis factor-α (TNF-α) through the TLR2-NF-κB signaling pathway, further activating the caspase-8-related pathway and promoting macrophage apoptosis, which kills intracellular *M. tuberculosis* (Basu et al., 2007; Green et al., 2011). In the early stages of infection, M1 macrophages are the main ones that exert the effect of anti-infective; they mainly induce reactive oxygen species bursts and release pro-inflammatory factors such as interleukin-6 (IL-6) to mediate Th1 type immune responses, which can directly killing *M. tuberculosis*. However, this can also cause tissue damage due to excessive immune damage. Some M2 macrophages also produce anti-inflammatory factors such as IL-4, mediating Th2 type immune responses to avoid excessive immune damage (Bain et al., 2009; Khan et al., 2019; Robinson et al., 2019). After identifying *M. tuberculosis* infection, TLR1/2 receptors are activated on the monocyte membrane and regulate the activation of vitamin D binding receptors (VDR), which then increases the secretion of antimicrobial protein (cathelicidin, LL37), exerting anti-infective effects (Liu et al., 2006). However, *M. tuberculosis* has evolved multiple strategies to evade the killing mechanisms of the host, such as inhibiting the fusion of phagosome-lysosome, which can help it survive in the phagosomes (Weng et al., 2023). In addition, other innate immune cells such as DC cells are also indispensable in the occurrence and development of the disease after *M. tuberculosis* infection, which can present antigens of *M. tuberculosis* to T lymphocytes and activate adaptive immune responses (Rodrigues et al., 2020).

As the infection of *M. tuberculosis* progresses, adaptive immune responses will be activated in order to clear the infection of *M. tuberculosis*, which mainly mediated by T cell and B cell subsets, such as CD4^+^ T cell, CD8^+^ T cells, γδ T cells and NK cells, et al (Chai et al., 2020). CD4^+^ T cells are the main antigen-specific cells that suppress *M. tuberculosis* infection, which play an important role in combating *M. tuberculosis* infection (Mayer-Barber and Barber, 2015). CD4^+^ T cells can secrete interferon-γ (IFN-γ), which not only enhances the activation of CD8^+^ T cells, but also slows down the depletion of CD8^+^ T cells, and further activates macrophages to enhance their bactericidal ability (Lu et al., 2021). After CD4^+^ T cells release IFN - γ, CD8^+^ T cells preferentially lyse antigen-presenting cells infected with *M. tuberculosis* through cytotoxic effects, which play an anti-infection monitoring role in this process (Lewinsohn et al., 2003). In addition, γδ T cells and NK cells also play a role in combating *M. tuberculosis* infection (Guerra et al., 2012; Liu et al., 2022). Studies have also found that CD4^+^ T cells and CD8^+^ T cells have the ability to clear *M. tuberculosis* from alveolar macrophages (Lai et al., 2024), further demonstrating the important role of T cells in combating *M. tuberculosis* infection. B cells often differentiate into different subsets in different micro-environments, such as Be1 and Be2 cells, and then produce different cytokines to regulate the maturation and differentiation of other immune cells, as well as the degree of inflammation in the body, achieving the goal of controlling *M. tuberculosis* infection (Shen and Fillatreau, 2015). In a word, the complex interaction between these immune cells and the perfect degree of mutual cooperation determines the outcome of the course of *M. tuberculosis* infection, whether it is controlled or develops into active pulmonary tuberculosis.

During the activation of innate and adaptive immune responses, many inflammatory factors such as TNF -α, IFN -γ, IL-12, and IL-1 β are released (Chandra et al., 2022), these inflammatory factors play a crucial role in the occurrence and development of tuberculosis. TNF-α plays an important role in the immune response of *M. tuberculosis* infection, which can activate macrophages, promote granuloma formation and induce apoptosis to participate in host defense, but excessive production of TNF-α may also lead to tissue damage (Beham et al., 2011; Kgoadi et al., 2025). IFN-γ is mainly produced by T cells and NK cells, which not only activates macrophages, but also enhances the anti-*M. tuberculosis* ability of macrophages (Kamboj et al., 2020). IL-12 plays an important role in connect between innate and adaptive immunity, which can promote the differentiation of Th1 cells and enhancing cellular immune response (Li et al., 2016). IL-1β participates in inflammatory processes, including fever, the acute phase response, and granuloma formation. The synergistic effects of TNF-α and IL-12 can enhance macrophage bactericidal activity, whereas IFN-γ and IL-1β jointly promote granuloma formation and maintenance, thereby preventing the dissemination of *M. tuberculosis* within the host. These inflammatory mediators interact through a complex network to regulate immune cell activity and function, influencing the progression and outcome of TB. Consequently, modulating the secretion of these cytokines may represent an effective strategy for controlling the development of TB.

## 3 Synthesis and metabolism of vitamin D

**Figure 2:**
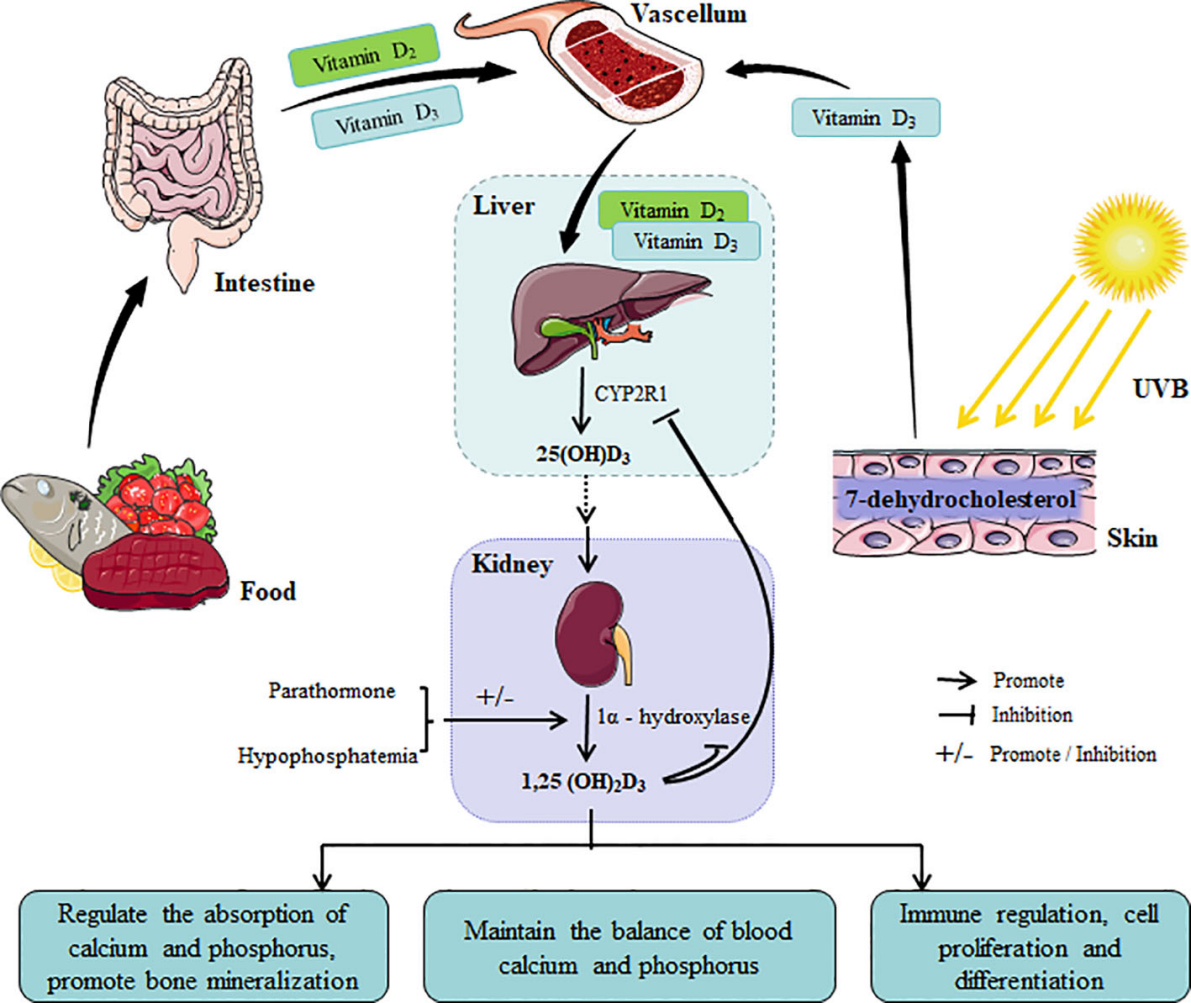
The synthetic metabolic pathway of vitamin D and its physiological functions. Vitamin D is derived from food and skin synthesis. After being absorbed in the small intestine, it is synthesized into active substances through negative feedback in parts such as the liver and kidneys, regulating the normal level of vitamin D in the human body to meet the functional needs of the body.

Vitamin D primarily exists in two forms: vitamin D_2_ (ergocalciferol) and vitamin D_3_ (cholecalciferol). These forms are crucial for maintaining normal physiological functions within the human body. Vitamin D deficiency can result in various disorders, including rickets in children and osteoporosis in adults (Holick and Chen, 2008). Vitamin D acquisition primarily occurs through dietary intake and cutaneous synthesis following UV irradiation. Dietary sources of vitamin D_3_ and vitamin D_2_ are predominantly animal and plant-based foods, respectively. 7-dehydrocholesterol in the skin serves as a precursor for vitamin D_3_ synthesis. However, vitamin D requires conversion to its active form to exert physiological effects. Active forms of vitamin D include 25-hydroxyvitamin D_3_ (25(OH)D_3_) and 1,25-dihydroxyvitamin D_3_ (1,25(OH)_2_D_3_), with 1,25(OH)_2_D_3_ being the primary active form. Dietary vitamin D is emulsified in the small intestine and absorbed into the bloodstream via bile. Both dietary and UV-synthesized vitamin D_3_ and vitamin D_2_ are transported to the liver, where they are converted to 25(OH)D_3_ by D25 hydroxylase (CYP2R1), the major storage and transport form of vitamin D in the body (Zhu et al., 2013; Liu and Leboff, 2024). Subsequently, 25(OH)D_3_ reaches the kidneys via circulation and interacts with 1α-hydroxylase within the mitochondria of renal proximal tubular epithelial cells. Regulated by parathyroid hormone, low serum phosphorus, and other factors, 1α-hydroxylase catalyzes the conversion of 25(OH)D_3_ to the highly active 1,25-(OH)_2_D_3_. As the active form of vitamin D, 1,25(OH)_2_D_3_ functions as a “messenger” binding to the VDR, which is highly expressed in various nucleated cells (immune cells, keratinocytes) and tissues (prostate, small intestine). This process regulates intestinal calcium and phosphorus absorption, promotes bone mineralization, maintains calcium and phosphorus homeostasis, and participates in physiological processes such as immune regulation, cell proliferation, and differentiation, thereby safeguarding human health (Chang and Lee, 2019; Cai et al., 2022). When the body has sufficient vitamin D, 1,25(OH)_2_D_3_ inhibits the activity of CYP2R1 in the liver and 1α-hydroxylase in the kidney via a negative feedback mechanism, thus preventing excessive vitamin D synthesis and activation, and maintaining vitamin D metabolic homeostasis (Jones et al., 2012) (**Figure 2**).

## 4 The treatment and mechanism of vitamin D in TB

In recent years, with the advancement of science and technology, numerous studies have indicated that vitamin D not only plays a crucial role in regulating blood calcium and phosphorus balance, as well as cell proliferation and differentiation, but also exhibits anti-viral (*Coronavirus*) (Ismailova and White, 2022), anti-bacterial (*Staphylococcus aureus*, *Pseudomonas aeruginosa*, and *Helicobacter pylori*) (Hosoda et al., 2015; Wang et al., 2015), and anti-fungal (*Candida albicans*) (Lei et al., 2022) effects. The anti-bacterial effect of vitamin D may be closely linked to its stimulation of antimicrobial peptide production in macrophages and the increased expression of VDR and PRRs in immune cells (Liu et al., 2006). Clinical research demonstrates that 25(OH)D_3_ in the serum of TB patients are generally low levels, and these low vitamin D levels correlate with the risk of TB, disease progression, and poor prognosis (Sato et al., 2012). Reports suggest that low vitamin D levels are prevalent in TB patients, and some studies have shown that vitamin D can enhance the immunity of TB patients in *in vitro* whole blood detection (Martineau et al., 2007; Nnoaham and Clarke, 2008). So, the potential role of vitamin D in TB patients treatment has garnered significant attention. Hui feng Yang et al. confirmed that vitamin D-deficient mice exhibit reduced immunity against *M. tuberculosis* infection (Yang et al., 2013), which aligns with previous findings indicating that individuals with low vitamin D levels are five times more likely to develop TB compared to those with normal levels (Talat et al., 2010). Furthermore, Zhang Jing et al. also confirmed in mice that the combination of pyrazinamide and vitamin D can inhibit the growth of *M. tuberculosis*, accelerate the resolution of lung lesions. Moreover, co-administration of vitamin D with pyrazinamide can also by regulating the balance between the pro-inflammatory and anti-inflammatory response and elevating the levels of antimicrobial peptides (increase the production of IL-4 and LL-37), thereby preventing excessive immune damage to tissues and organs while achieving the goal of clearing *M. tuberculosis* infection (Zhang et al., 2019). The therapeutic effect of vitamin D in *M. tuberculosis* has been studied not only in mice, but also in humans (**Table 1**), in a clinical controlled trial, it was found that the treatment time of TB patients was significantly shortened after vitamin D supplementation (Eletreby et al., 2024). Cussens AK et al. found that vitamin D may help tuberculosis treatment by slowing down the inflammatory reaction related to the increased risk of death (Coussens et al., 2012). A study found that adding vitamin D as adjuvant therapy in patients with advanced pulmonary tuberculosis not only significantly increased sputum conversion rate, but also improved lung radiographical (Nursyam et al., 2006), the same effect has been achieved in patients with diabetes and pulmonary tuberculosis (Kota et al., 2011). And research has also found that supplementing with higher doses of vitamin D (oral 2.5 mg/day or intramuscular injection 600,000 IU) has a good adjuvant therapeutic effect on the treatment of *Mycobacterium tuberculosis*, while supplementing with low-dose vitamin D (intramuscular injection 100,000 IU) has no significant effect (Wejse et al., 2009; Martineau et al., 2011; Salahuddin et al., 2013), which suggest that vitamin D predicts TB disease risk in a dose-dependent manner (Aibana et al., 2019). However, more study is needed to confirm the dosage of vitamin D for TB patients.

**Table 1:** Studies on the therapeutic effects of vitamin D on tuberculosis in clinical trials.

Pulmonary TB/extrapulmonary TB	Conclusion	Administration	Dosage	Mode of intervention	References
Pulmonary TB	Vitamin D can serve as adjuvant treatment of tuberculosis in diabetics with vitamin D deficiency	Take orally	Vitamin D3 (60,000 units/week) and calcium carbonate (1g/day)	Part of ATT	(Kota et al., 2011)
	Vitamin D supplementary treatment can significantly increase the improvement of radiological results in TB patients	Take orally	Vitamin D (0.25 mg/day)	Single supplementary	(Nursyam et al., 2006)
	Supplementation with high doses of vitamin D accelerated clinical, radiographic improvement in all TB patients	Intramuscular	Vitamin D3 (600,000 IU/400,000 IU for 2 doses/per treatment cycle)	Single supplementary	(Salahuddin et al., 2013; Hussain et al., 2023)
	Adding vitamin D as adjuvant therapy for pulmonary TB significantly improved the conversion rate of sputum culture in patients	Take orally	Vitamin D3 (2·5 mg for 3 doses/per treatment cycle); calcitriol (0.25 µg/day)	Single supplementary/ Part of ATT	(Martineau et al., 2011; Wen et al., 2022)
	Vitamin D supplementation significantly improved the symptoms of pulmonary tuberculosis treatment	Take orally	Vitamin D (400 IU/day)	Part of ATT	(Wang et al., 2020)
	Vitamin D augmented ATT can be more efficacious in treating patients with pulmonary TB compared to the standard ATT	Take orally	Vitamin D3 (600 IU/day)	Part of ATT	(Hasanain et al., 2019)
Extrapulmonary TB	vitamin D supplementation is a useful adjunctive therapy to anti-TB drugs and improves treatment course in extrapulmonary TB	Take orally	Vitamin D3 (50,000 IU/week for 6 weeks)	Part of ATT	(Eletreby et al., 2024)
	Vitamin D deficiency was an independent predictor of extrapulmonary tuberculosis	–	–	–	(Hammami et al., 2021) ^※^

ATT, Anti-tuberculosis therapy; “-’’: Without; “※”: Case-control study, which conclusions are drawn by analyzing and collecting data.

Presently, research on the role of vitamin D in the potential treatment of tuberculosis is more focused on the cellular level, such as macrophages, THP-1 cells, monocytes, and DC cells. It has been confirmed that vitamin D not only inhibits *M. tuberculosis* infection in macrophages by inducing autophagy (Campbell and Spector, 2012), but is also necessary for IFN-γ to mediate the antibacterial activity of human macrophages (Fabri et al., 2011). Vitamin D directly acts on *M. tuberculosis* by increasing the expression of LL37 in macrophages, disrupting the cell wall/membrane of *M. tuberculosis*, and inhibiting the concentration of Ca^2+^ to activate autophagy in THP-1 cells infected with *M. tuberculosis*, in order to achieve the goal of clearing *M. tuberculosis* (Wu et al., 2022). It also regulates the polarization of monocytes, which can control the infection of *M. tuberculosis* (Rao Muvva et al., 2019). Vitamin D regulates the expression of LL37 in DC cells, promoting Th1 cell differentiation, inhibiting Th17 immune response, and enhancing the ability to combat the infection of *M. tuberculosis* (Rode et al., 2017). Susana Flores Villalva et al. found that 1,25(OH)_2_D_3_ increases the clearance ability of *Bovine Tuberculosis Bacill*i by stimulating neutrophils to increase the production of ROS (Flores-Villalva et al., 2023). In addition, studies have found that individuals carrying the Fok1 polymorphism FF genotype have better therapeutic effects on vitamin D treatment compared to other genotypes of tuberculosis patients (Bavi et al., 2024). Jiezhong Deng et al. found that vitamin D can inhibit the activity of the NF-κB signaling pathway, which can reduce the generation of osteoclasts, and achieve the goal of inhibiting *M. tuberculosis*-induced bone destruction (Deng et al., 2021), which indicate that vitamin D not only enhances the ability of immune cells to combat *M. tuberculosis* infection, but also plays an important role in osteoclast resistance to *M. tuberculosis* infection, this is of great significance for the treatment of bone destruction caused by *M. tuberculosis*. This indicates that vitamin D has immunomodulatory effects on both innate and adaptive immune cells and is of great significance in clearing *M. tuberculosis*.

The dysregulation of inflammatory factors in tuberculosis patients can easily lead to immunopathological damage, such as lung tissue destruction or cavity formation. Therefore, elucidating the mechanism of inflammatory factors in TB patients may be crucial for developing novel treatment strategies. Yiming Wu et al. found that vitamin D significantly reduced cellular damage in the lung tissue of mice infected with *M. tuberculosis* (Wu et al., 2022). In a randomized, placebo-controlled study of 200 TB patients, it was found that, compared with TB patients receiving placebo treatment, patients receiving vitamin D showed significant differences in weight gain, improved lung imaging results, and an increase in IFN-γ levels *in vivo* (Hussain et al., 2023), indicating that vitamin D supplementation has a beneficial therapeutic effect in the treatment of TB. As an important active substance of vitamin D, 1,25-(OH)_2_D_3_ is a potent immunomodulatory agent that exerts its effects by binding to the VDR in the cell nucleus. Previous studies have confirmed that after administration of 1,25 (OH)_2_D_3_, T cells exert anti-inflammatory effects by downregulating the levels of MCP-1, MIP-1β, and IP-10 in the serum (Harishankar et al., 2016). 1,25 (OH)_2_D_3_ can downregulate the content of matrix metalloproteinases (MMPs) and upregulate the tissue inhibitors of metalloproteinases (TIMPs) in cell culture supernatant after co-culture with peripheral blood mononuclear cells for 48 hours, thus playing a role in tissue remodeling in TB patients (Anand and Selvaraj, 2009). Furthermore, by reducing the expression of *TLR2*, *TLR4*, *Dectin-1*, and mannose receptors, the mRNA levels of pro-inflammatory cytokines *IL-6*, *TNF-α*, and *IFN-γ* were down-regulated, while the production of IL-10 and LL37 were up-regulated (Khoo et al., 2011). In addition, 1,25 (OH)_2_D_3_ can also promote the release of cytokines such as IL-1β, IL-10, TNF-α, and IL-12p40 in monocyte-derived macrophages and enhance the ability of human monocyte-derived macrophages in activeTB patients to restrict the growth of *M. tuberculosis* (Eklund et al., 2013). The inflammatory response is a double-edged sword, an appropriate inflammatory response can help control infection, but an excessive inflammatory response may lead to tissue damage and disease deterioration. The anti-inflammatory effect of vitamin D and its restrictive effect on the growth of *M. tuberculosis* are of great significance in reducing inflammation and tissue damage during disease activity and controlling the spread of *M. tuberculosis* (Papagni et al., 2022; Chandra et al., 2024).

Early research in 1986 demonstrated that vitamin D treatment of macrophages significantly inhibited *M. tuberculosis* growth (Rook et al., 1986). Current investigations into vitamin D’s anti-mycobacterial activity primarily emphasize its indirect enhancement of host immune function. However, prior studies have validated vitamin D’s direct inhibitory effects on *M. tuberculosis*. In 2012, Greenstein et al. confirmed the direct inhibition of *M. tuberculosis* growth by vitamin D *in vitro* (MIC = 64 μg/mL) (Greenstein et al., 2012). However, further research is warranted to elucidate the mechanism of vitamin D’s *in vitro* inhibitory effects on *M. tuberculosis*.

## 5 Discussion and prospect

This review summarizes the immune response to *M. tuberculosis* infection, followed by an overview of vitamin D synthesis and metabolism, and explores the potential mechanisms of vitamin D in combating *M. tuberculosis* infection. In recent years, an increasing number of studies have found that vitamin D not only has a good preventive effect on the occurrence of chronic diseases, but also promotes the release of anti-inflammatory cytokines at high vitamin D levels (Argano et al., 2023). In addition, the discovery of good antibacterial effects of vitamin D on *Staphylococcus aureus, Pseudomonas aeruginosa, Helicobacter pylori* and *Candida albicans* suggests that vitamin D may be a potential candidate for the development of drugs against *M. tuberculosis*. Current reports on vitamin D’s anti-tuberculosis effects primarily focus on its ability to modulate host immune function, indirectly exerting its anti-tuberculosis effects. The potential mechanisms of vitamin D’s anti- *M. tuberculosis* effects include: 1) Regulation of immune cell function; 2) Modulation of the inflammatory response; 3) Direct inhibitory effects on *M. tuberculosis*. However, there is a paucity of research on the direct inhibitory effects of vitamin D on *M. tuberculosis* growth *in vitro* and *in vivo*, and the mechanisms underlying these effects. While *in vitro* studies have demonstrated that vitamin D activates immune cells and exerts anti-inflammatory effects by regulating inflammatory cytokine secretion to limit *M. tuberculosis* spread *in vivo*, *in vivo* experiments have largely been limited to the animal level, with limited efficacy in clinical trials. Some studies have even reported no significant difference between TB patients treated with vitamin D and the control group (Ganmaa et al., 2020). Therefore, the role of vitamin D in TB remains debated. Potential factors contributing to these discrepancies include insufficient sample sizes, inaccurate data, comorbidities, variations across countries, regions, or genders, inconsistencies in patient immune status, uncertainty regarding optimal vitamin D supplementation levels, and potential metabolic interactions between anti-*M. tuberculosis* drugs and vitamin D, all of these issues warrant further investigation.

Despite these limitations, the role of vitamin D in TB prevention is significant. Sufficient vitamin D levels can augment host immune defenses, thereby mitigating the risk of *M. tuberculosis* infection. Vitamin D supplementation may be an effective preventative strategy, particularly in high-risk regions and susceptible populations (Aibana et al., 2019). The potential adjuvant role of vitamin D in TB treatment has been preliminarily confirmed, suggesting broad application prospects. Future research should elucidate the mechanisms of vitamin D in TB treatment, optimize clinical application protocols, and investigate its synergy with other anti-tubercular drugs. With further research and clinical experience, vitamin D is poised to become a crucial adjunct in TB treatment, playing a more significant role in global TB prevention and control, and offering novel strategies and methodologies.
